# Ecological Insights Into Community Interactions, Assembly Processes and Function in the Denitrifying Phosphorus Removal Activated Sludge Driven by Phosphorus Sources

**DOI:** 10.3389/fmicb.2021.779369

**Published:** 2021-11-10

**Authors:** Lei Zheng, Xue Wang, Aizhong Ding, Dongdan Yuan, Qiuyang Tan, Yuzi Xing, En Xie

**Affiliations:** ^1^College of Water Sciences, Beijing Normal University, Beijing, China; ^2^College of Water Resources and Civil Engineering, China Agricultural University, Beijing, China

**Keywords:** activated sludge, phosphorus, microbial assembly, network structures, metabolic function

## Abstract

The microbial characteristics in the wastewater treatment plants (WWTPs) strongly affect their optimal performance and functional stability. However, a cognitive gap remains regarding the characteristics of the microbial community driven by phosphorus sources, especially co-occurrence patterns and community assembly based on phylogenetic group. In this study, 59 denitrifying phosphorus removal (DPR) activated sludge samples were cultivated with phosphorus sources. The results suggested that homogeneous selection accounted for the largest proportion that ranged from 35.82 to 64.48%. Deterministic processes dominated in 12 microbial groups (bins): *Candidatus_*Accumulibacter and *Pseudomonas* in these bins belonged to phosphate-accumulating organisms (PAOs). Network analysis revealed that species interactions were intensive in cyclic nucleoside phosphate-influenced microbiota. Function prediction indicated that cyclic nucleoside phosphates increased the activity of enzymes related to denitrification and phosphorus metabolism and increased the α-diversity of microorganism but decreased the diversity of metabolic function. Based on these results, it was assumed that cyclic nucleoside phosphates, rather than inorganic phosphates, are the most available phosphorus source for majority microorganisms in DPR activated sludge. The study revealed the important role of phosphorus source in the construction and assembly of microbial communities and provided new insights about pollutant removal from WWTPs.

## HIGHLIGHTS

- P could significantly influence microbial community assembly processes in DPR system
- Bacterial interplays were more intensive in cNMPs influenced community
- cNMPs increased species diversity and decreased metabolic function diversity
- cNMPs increased the activity of enzymes related to denitrification and P metabolism

## Introduction

Efficient wastewater treatment technologies are fundamental for reducing nutrient emissions (particularly nitrogen and phosphorus) in the water bodies and to cope up with serious eutrophication (Wurtsbaugh et al., 2019). Denitrifying phosphorus removal (DPR) process has gained growing attention, as it solves the problem of carbon source competition encountered in traditional nitrogen and phosphorus removal processes (Liu et al., 2014). In the process of nitrogen and phosphorus removal, nutrients are the key factors affecting the system performance (Chen et al., 2017). The lack of suitable phosphorus source in the influent can easily result in sludge bulking and inhibit the proliferation of denitrifying polyphosphate (poly-P)-accumulating organisms (DPAOs) and then affect the system function (Zhang et al., 2017). With the development of molecular biology, phylogenetic investigation of communities by reconstruction of unobserved states 2 (PICRUSt2) (Douglas et al., 2020), which is based on 16S rRNA sequencing data, can predict the functional part of metagenome through marker gene data and databases of reference genome, so as to find the direct evidence of the functional ability of microbial community (Huang et al., 2019). With PICRUSt2, the metabolic capacity of nitrogen and phosphorus can be anticipated, as well as the factors affecting the efficiency of nitrogen and phosphorus removal in DPR can be determined (Wang Z.B. et al., 2021).

The collective activities of various core functional taxa determine the ideal performance of the DPR process (Wang R. et al., 2021). In this process, various functional taxa cooperate with each other in the form of a complex network to achieve functions such as nitrogen and phosphorus removal that cannot be achieved by a single species (Yin et al., 2021). Moreover, these microorganisms vary in the utilization of inorganic and organic phosphorus; some microorganisms can form poly-P and then become dominant species (Huang et al., 2015). Recent studies have mostly focused on the competition between DPAOs and glycogen-accumulating organisms (GAOs), and on the richness or composition of DPR microbial communities (Izadi et al., 2020). Network analysis, thus, can add an important dimension to understand the microbial community of DPR process. In recent years, the microbial co-occurrence network has been widely used to unravel the microbial–microbial relationship in complex environments such as the ocean (Faust et al., 2018), soils (Tan et al., 2020), and activated sludge (Lin et al., 2021). Therefore, understanding the microbial interactions in the DPR system and their response to various phosphorus sources is very important for formulating an operational strategy to improve the performance of DPR process.

The study of species co-occurrence patterns in activated sludge systems can improve our understanding of the composition and diversity of microbial communities (Dal Co et al., 2020). However, there has been much controversy about the research of community assembly mechanism (Zhou and Ning, 2017; Liebana et al., 2019; Zhang et al., 2019). It has been recognized that deterministic process (including heterogeneous selection and homogeneous selection) plays a major role in bioreactor microbial assembly in some cases (Vanwonterghem et al., 2014; Xu et al., 2020), but in other cases, stochastic process (including homogenizing dispersal, dispersal limitation, and drift) dominate (Zhou et al., 2013; Xu et al., 2019). However, previous studies on assembly mechanisms have focused on evaluation at the level of the “whole community.” It is widely known that microbial communities are usually composed of different interacting genotypes, which are non-randomly arranged in space, called spatial self-organization (Lion and van Baalen, 2008). The difference in the local initial spatial positions of the microbes promotes the diversification of spatial self-organization patterns (Goldschmidt et al., 2021). The microbial communities with highly diverse spatial self-organization patterns tend to have diverse community properties, where individuals interact with each other during their growth and division (Dal Co et al., 2020). Thus, there are reasons to believe that different “microorganism groups” in activated sludge have different assembly mechanisms. However, there are still some knowledge gaps in the research of assembly mechanism based on phylogenetic groups. Recently, inferring community assembly mechanisms by phylogenetic-bin-based null model [Infer Community Assembly Mechanisms by Phylogenetic-bin-based null model (iCAMP)] was to infer the community assembly mechanism, which is considered to be an assembly mechanism at the level of a single taxon/pedigree, rather than the entire community (Ning et al., 2020). This framework can reveal the community assembly mechanism of different microbial communities in DPR activated sludge driven by phosphorus sources from a level of a single taxon/pedigree.

In this study, the analyses of microbial diversity, community assembly process based on null model, co-occurrence network analysis, and prediction of key metabolic pathways were used to investigate the effect of phosphorus on microbial assembly in the DPR systems. Specifically, this study addressed three main points: (1) the microbial community structure and species interactions in the DPR influenced by four classes of phosphorus sources; (2) the driving processes that regulate the bacterial community assembly in DPR process; and (3) nitrogen and phosphorus metabolism in microorganisms under the influence of different phosphorus sources.

## Materials and Methods

### Experimental Design and Operation

The activated sludge was procured from an operating activated sludge reactor at Beijing Normal University and was then cultured in sequencing batch reactor (SBR) for 90 days at room temperature. The design and operation were modified based on our previous research, and the details can be found in Zheng et al. (2019). Briefly, the SBR operation pattern can be found in Supplementary Method 1. Influent was municipal wastewater taken from the campus of Beijing Normal University, and its detailed characteristics are shown in Supplementary Table 1. In order to acclimatize the activated sludge with nitrate as electron acceptor, NaNO_3_ was added in the anoxic section. The dosage of NaNO_3_ was 60.70 mg/L (10.00 mg N/L) in the first 15 days and 151.80 mg/L (25.00 mg N/L) in the last 25 days of reactor operation. When the effluent quality became stable, and mixed liquor suspended solid (MLSS) of activated sludge reached 3.50 ± 0.10 g/L, the activated sludge was transferred into a 96-well cell-culture microplate, and phosphorus-free M9 medium (Supplementary Method 2) was used to cultivate the activated sludge, in which pH was adjusted to about 7.0 and was autoclaved. To achieve a P concentration of 15.5 mg/L, 0.01 mmol (calculated as P) of 59 types of the most common phosphorus sources was selected to add to the M9 medium per well. According to phosphorus chemical structure, these were divided into four categories: seven inorganic phosphates (IPs), nine cyclic nucleotide monophosphates (cNMPs), 14 nucleoside monophosphates (NMPs), and 29 other organophosphates (OPs). Specific classification of these phosphorus sources is presented in detail in Supplementary Table 2. Then the cell-culture microplate was cultured in an incubator (HPS-250, Donglian Electronic Technology Development Company, Harbin, China) at 30°C for 60 h.

### DNA Extraction and High-Throughput Amplicon Sequencing

The sludge samples were collected from cell-culture microplate after culture for 60 h, and each sample had three replicates. TIANamp Bacteria DNA Kit (Tiangen Biotech, Beijing, China) was used to extract DNA from activated sludge and subjected to bacterial V3–V4 region amplification using primers 338F (5′-ACTCCTACGGGAGGCAGCAG-3′) and 806R (5′-GGACTACHVGGGTWTCTAAT-3′). Libraries were sequenced on the Illumina MiSeq platform with a Paired-End protocol. More detailed descriptions on DNA extraction and bacterial region amplification are available in Zheng et al. (2019). The raw reads were deposited into the National Center for Biotechnology Information (NCBI) Sequence Read Archive database under accession PRJNA723985.

### Microbial Community Analysis

At the lowest sequencing depth of 17,144, the α-diversity analysis and rarefaction curves of bacterial community were estimated by Liu et al. (2020). The difference of bacterial community and functional structure was characterized by principal coordinate analysis (PCoA) based on the Bray–Curtis distance. With the use of linear discriminant analysis (LDA) ≥ 3.5, LDA effect size (LEfSe) analysis was conducted to detect potential bacterial biomarkers^1^. The LDA score from the LEfSe analysis was used to show the relationship between taxa using a cladogram (circular hierarchical tree).

The phylogenetic molecular ecological networks (pMENs) of the bacteria communities were constructed on a comprehensive molecular ecological network analysis pipeline (MENA^2^) (Deng et al., 2012), and Gephi (0.9.2) (Bastian et al., 2009) was used for visualization. Among-module connectivity (*P*_*i*_) and within-module connectivity (*Z*_*i*_) were calculated to determine the node connectivity (Olesen et al., 2007).

The assembly mechanism of different microorganism groups was investigated by the iCAMP. The calculation of iCAMP was analyzed using an in-house Galaxy software platform (IEG Statistical Analysis Pipeline^3^) (Ning et al., 2020). The difference of community value between the two groups was measured by PERMANOVA test (*n* = 999).

According to the 16S rRNA marker gene profiles, PICRUST2 was used to predict the functional composition of microbial community (Douglas et al., 2020). The functional annotation of PICRUST2 prediction was obtained based on the Kyoto Encyclopedia of Genes and Genomes (KEGG) database. The table of KEGG orthologs (KOs) was generated for each data set, and the predicted relative abundance of KOs was calculated.

### Chemical and Statistical Analyses

Standard methods were used to measure the MLSS of the activated sludge and to measure the NO_3_^–^–N, TN, TP, and orthophosphate of the in- and out-fluent (Federation and Association, 2005). After the sludge was cultured in M9 medium for 60 h, the NO_3_^–^–N, total organic carbon (TOC), alkaline phosphatase (AKP) activities, and orthophosphate were determined. AKP activity was evaluated according to the EnzChek Phosphatase Assay kit (Molecular Probes, Eugene, OR, United States) operating instructions (Chen et al., 2016). TOC was evaluated by TOC analyzer (vario TOC cube, Elementar, Langenselbold, Germany). Spearman’s correlation and Kruskal–Wallis test analysis were performed using IBM SPSS Statistics (v 20.0) (SPSS, Chicago, IL, United States). *p*-Value < 0.05 was considered statistically significant.

## Results

### Impact of Phosphorus Source on the Performance of Denitrifying Phosphorus Removal System

**FIGURE 1:**
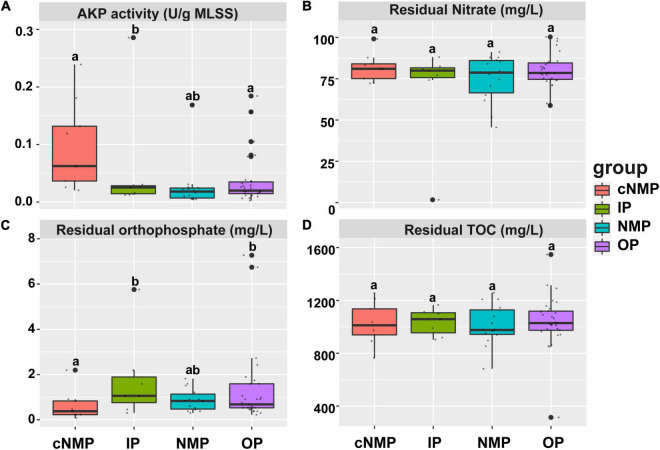
**(A)** Relative AKP activity, **(B)** residual nitrate, **(C)** residual orthophosphate, and **(D)** residual TOC of DPR with four phosphorus sources after 60 h of cultivation. The top and bottom boundaries of each box represent the 75th and 25th quartile values, respectively; and the line within each box represents the median values (*n* = 9, 7, 14, and 29). Different letters indicate significant differences at the *p* < 0.05 level. AKP, alkaline phosphatase; TOC, total organic carbon; DPR, denitrifying phosphorus removal.

Four different types of phosphorus sources were used to study their impact on the residual orthophosphate, TOC, nitrate, and AKP activity of 59 sludge samples. It was observed that the phosphorus sources significantly affected the AKP activity and utilization of orthophosphate in DPR system, while no significant difference in the carbon and nitrogen removal efficiencies was observed (Figure 1). Concretely, sludge cultivated with IPs had higher residual orthophosphate and lower AKP activity than that cultivated with cNMPs (*p* < 0.05, Figures 1A,C). In Figure 1C, the outlier in IPs of residual orthophosphate was orthophosphate, with its concentration up to 5.76 mg/L higher than other IPs. The sludge with higher residual orthophosphate concentration cultivated by cNMPs, IPs, NMPs, and OPs tended to show lower AKP activity, and there was a significant negative correlation between residual orthophosphate concentration and AKP activity (*r* = −0.570, *p* < 0.05). This might be attributed to the presence of orthophosphate, which can inhibit the expression of AKP coding genes, thus reducing AKP activity (Ternan et al., 1998). These results indicated that different types of phosphorus sources can affect the enzymatic activities related to DPR by affecting the concentration of orthophosphate.

### Bacterial Diversity and Composition of Microbial Community With Different Phosphorus Sources

**FIGURE 2:**
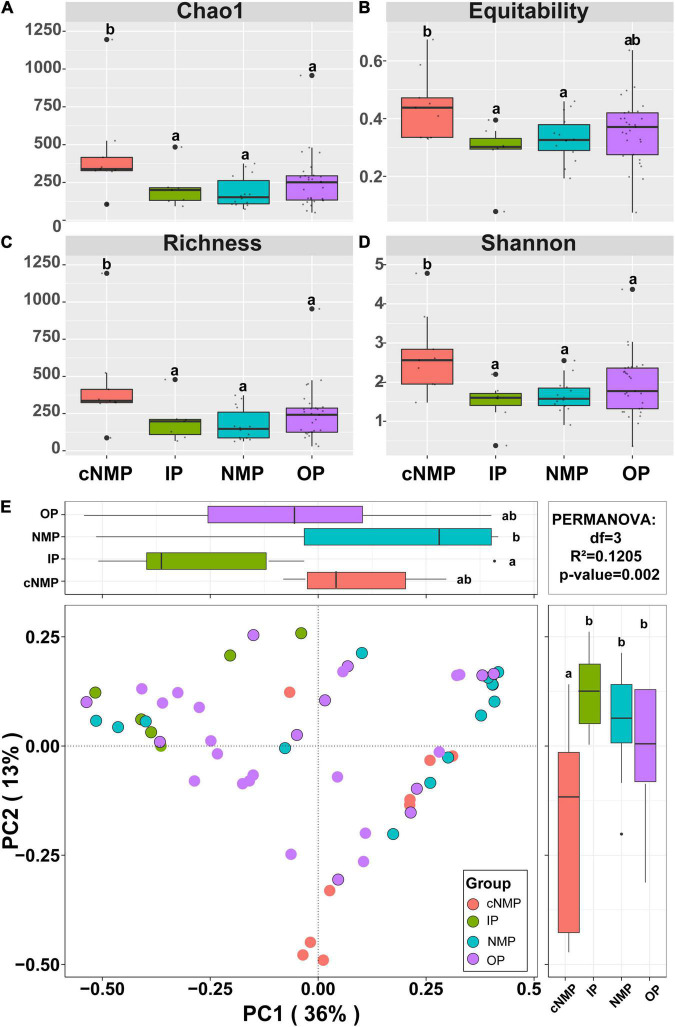
Community species compositions and diversity of bacterial community in DPR with different phosphorus sources. Species diversity characterized by the **(A)** Chao1 index, **(B)** Equitability index, **(C)** Richness index, and **(D)** Shannon index. **(E)** Ordination of bacterial community compositions (at OTU level) by PCoA based on the Bray–Curtis dissimilarity matrix. Different letters indicate significant differences at the *p* < 0.05 level. DPR, denitrifying phosphorus removal; OTU, operational taxonomic unit; PCoA, principal coordinate analysis.

A total of 2,202,777 high-quality filtered sequences were retrieved from 59 sludge samples, resulting in 2,330 operational taxonomic units (OTUs). Rarefaction curves of OTUs similarity-based at 97% sequence similarity level indicated that each sample had a majority of bacterial diversity (Supplementary Figure 1). Within-sample diversity (α-diversity) revealed a significant difference between IPs and cNMPs (*p* < 0.05) (Figures 2A–D). Here, the bacterial community with cNMPs showed higher diversity than with IPs, indicating that cNMPs recruited more bacterial species than IPs. However, OPs and NMPs showed no significant difference in α-diversity as compared with IPs (*p* > 0.05) (Figures 2A–D), which may be because the community structure with OPs and NMPs was similar to that with IPs (Figures 2A–D). PCoA based on the Bray–Curtis distances indicated that the bacterial community structures in DPR system shifted under different phosphorus sources (Figure 2E). The PCoA diagram explained 49% of all the differences observed in the sample. IP and NMP groups showed the most significant differences in the microbiome (*p* < 0.05), which can be well explained by the first axis. cNMPs also showed significantly different microbiome when compared with IPs, which is the second major reason of the different microbial structures in the overall experiment and can be well distinguished on the second sitting axis (*p* < 0.05) (Figure 2E and Supplementary Table 3).

**FIGURE 3:**
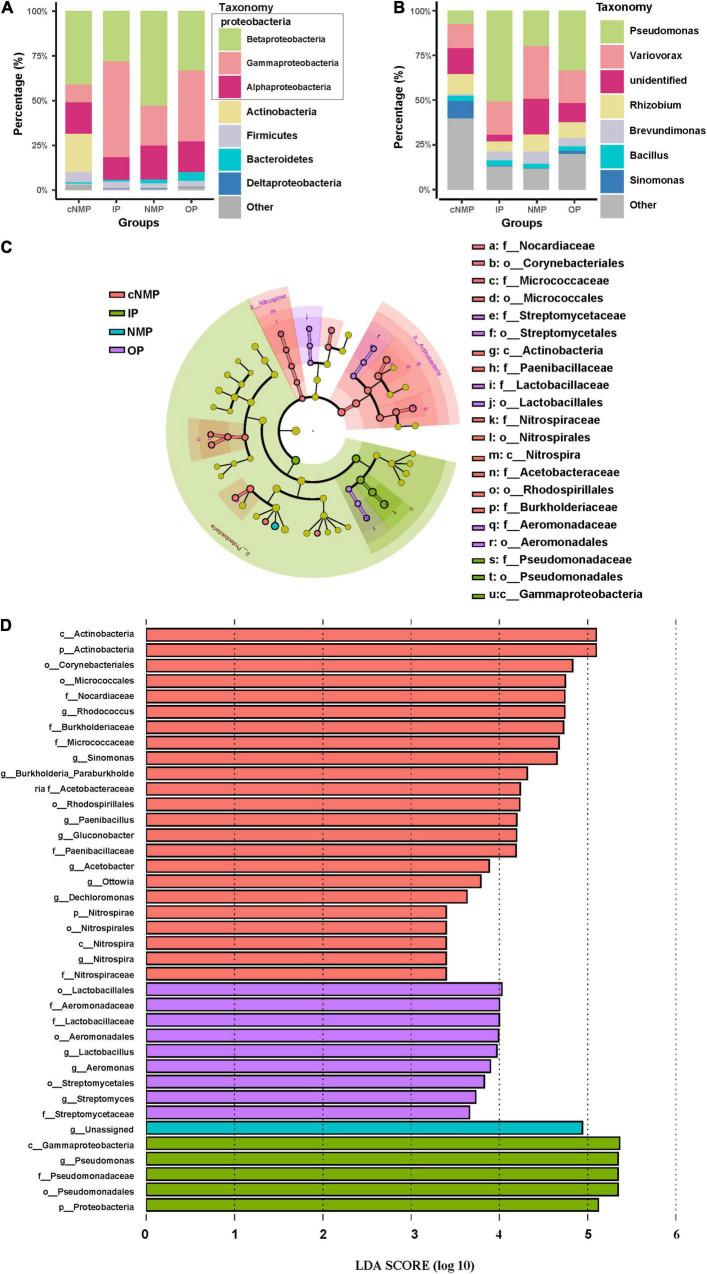
Relative abundance at **(A)** the phylum level (Proteobacteria at the class level) and **(B)** the genus level in DPR inoculated with different phosphorus sources. Taxonomic differences of DPR microbiota between four phosphorus sources from the LefSe method. **(C)** Taxonomic cladogram. The taxa with enrichment levels in different phosphorus sources are displayed in various colors. **(D)** The LDA scores observed for individual taxa that passed the LefSe significance threshold. The three species at the class level that belonged to Proteobacteria are shown in gray box in panel **(A)**. DPR, denitrifying phosphorus removal; LefSe, linear discriminant analysis effect size; LDA, linear discriminant analysis.

Proteobacteria was the most abundant phylum in DPR, accounting for 69.27–94.39% of the total bacterial sequences. Within Proteobacteria, the major classes of bacteria observed when supplemented with IPs, OPs, and NMPs were Betaproteobacteria and Gammaproteobacteria. With IPs, these classes accounted for 81.67 ± 7.23%; with NMPs, these accounted for 75.14 ± 7.85%; while with OPs, these accounted for 72.81 ± 9.58% of the total bacterial abundance. With cNMPs, a low abundance of 51.11% for the two classes of bacteria was observed (Figure 3A). At the genus level, *Pseudomonas* and *Variovorax* were predominant in samples with IPs, OPs, and NMPs. On the other hand, no significantly predominant genus was observed in cNMP samples, which reflected the higher equitability in these samples (Figures 2B, 3B). To find the biomarkers of DPR, LEfSe analysis of the microbial community was done under the action of four types of phosphorus sources (Figures 3C,D). The LEfSe evaluation identified *Pseudomonas*, belonging to Gammaproteobacteria, as the potential biomarker of sample with IPs. For cNMP-containing samples, *Rhodococcus* and *Sinomonas* affiliated with Actinobacteria; *Paenibacillus* affiliated with Bacilli; *Gluconobacter* and *Acetobacter* affiliated with Alphaproteobacteria; *Ottowia*, *Dechloromonas*, *Burkholderia*, and *Paraburkholderia* affiliated with Betaproteobacteria; and *Nitrospira* affiliated with *Nitrospira* were identified as the potential biomarkers (Figures 3C,D). The biomarkers in OP-containing samples were *Lactobacillus* affiliated with Bacilli; *Aeromonas* affiliated with Gammaproteobacteria; and Streptomycetales affiliated with Actinobacteria. The NMP-containing sample had only one biomarker, Comamonadaceae, affiliated with Betaproteobacteria (Figures 3C,D). Among these biomarkers, *Pseudomonas*, *Aeromonas*, *Rhodococcus*, *Sinomonas*, and *Paenibacillus* are capable of phosphorus accumulation as poly-P (Sidat et al., 1999; Hernandez et al., 2008; Alori et al., 2017). *Ottowia* and *Dechloromonas* are mainly involved in the degradation of organic substrates and nitrogen removal in the denitrification process (Liu et al., 2017). In activated sludge, *Nitrospira* is the most dominant nitrite-oxidizing bacteria among the nitrifying bacteria (Irie et al., 2016; Wang et al., 2017). The different types of phosphorus sources may change the microbial community structure and diversity in DPR.

### Impact of Phosphorus Sources on the Relative Importance of Deterministic and Stochastic Processes

In view of the changes in the microbial diversity and community composition of DPR activated sludge driven by phosphorus sources, the driving force that shapes the DPR sludge community structure, that is, the community assembly mechanism, was studied. The community assembly mechanism is the theoretical basis for monitoring activated sludge through the operating parameters of wastewater treatment plants (WWTPs) (Zhang et al., 2020). From an ecological point of view, the deterministic process included heterogeneous selection and homogeneous selection, while the stochastic processes included homogenizing dispersal, dispersal limitation, and drift (Zhou and Ning, 2017). In activated sludge, some phylogenetic taxa are in a state of strong selection, while others may be in a state of strong drift (Sun et al., 2021). This study used the newly developed tool iCAMP to study the community assembly mechanism of diverse microbial groups (called “bins”) in DPR activated sludge. Here, the regional species pool included the total number of all OTUs found in all 59 samples driven by four classes of phosphorus sources.

**FIGURE 4:**
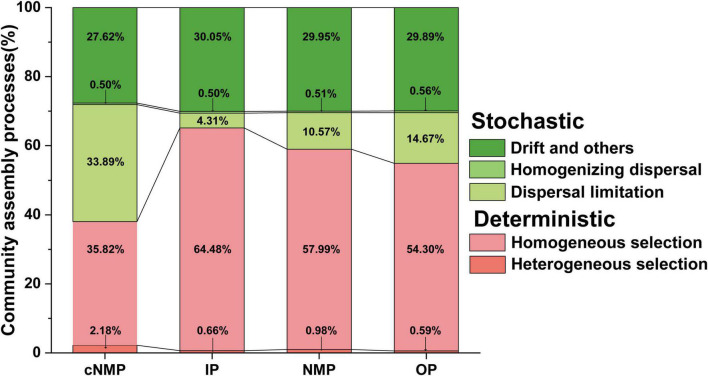
The relative contribution of deterministic and stochastic processes under the change of bacterial community assembly in DPR with different phosphorus sources. Deterministic included heterogeneous selection and homogeneous selection. Stochastic included drift and others, homogenizing dispersal, and dispersal limitation. DPR, denitrifying phosphorus removal.

The tool distributed 2,330 observed OTUs to 42 phylogenetic bins. Specifically, homogeneous selection accounted for the largest proportion of DPR activated sludge assembly mechanism (cNMPs, 35.82%; IPs, 64.48%; NMPs, 57.99%; and OPs, 54.30%), and the homogeneous selection in community driven by cNMPs was significantly lower than that in IPs, NMPs, and OPs (*p* < 0.05; Figure 4 and Supplementary Table 4B). Drift and others and dispersal limitation were followed, and dispersal limitation had a maximum influence on the community in samples containing cNMPs (33.89%), while the influence on other three communities was lower (IPs, 4.31%; NMPs, 10.57%; and OPs, 14.67%) (*p* < 0.05; Figure 4 and Supplementary Table 4C). Homogenizing dispersal (cNMPs, 0.50%; IPs, 0.50%; NMPs, 0.51%; and OPs, 0.56%) and heterogeneous selection (cNMPs, 2.18%; IPs, 0.66%; NMPs, 0.98%; and OPs, 0.59%) accounted for smaller proportions (Figure 4).

The deterministic processes in DPR activated sludge community assembly dominated in 12 bins (bin numbers, 28.57%; relative abundance, 68.22%), while the stochastic processes in DPR activated sludge community assembly dominated in 30 bins (bin numbers, 71.43%; relative abundance, 31.78%). Homogeneous selection was predominant in 12 bins. Thus, the 12 bins in community assembly were selected under the selection pressure of phosphorus sources. The classification information of these 12 bins was mostly affiliated to Planctomycetes, Firmicutes, Actinobacteria, Gemmatimonadetes, Acidobacteria, Proteobacteria, and Bacteroidetes (Supplementary Table 5). *Candidatus_*Accumulibacter and *Pseudomonas* in bin_31_ and bin_32_ appeared as deterministic bins in the community assembly driven by cNMPs, IPs, and OPs. In addition, *Candidatus_*Accumulibacter and *Pseudomonas* belonged to phosphate-accumulating organisms (PAOs). These microbial groups that can be selected by DPR sludge for the deterministic assembly process play an important role and may be the basis for activated sludge community regulation, and their functions need to be further studied.

### Network Topology Features of the Bacterial Community

**FIGURE 5:**
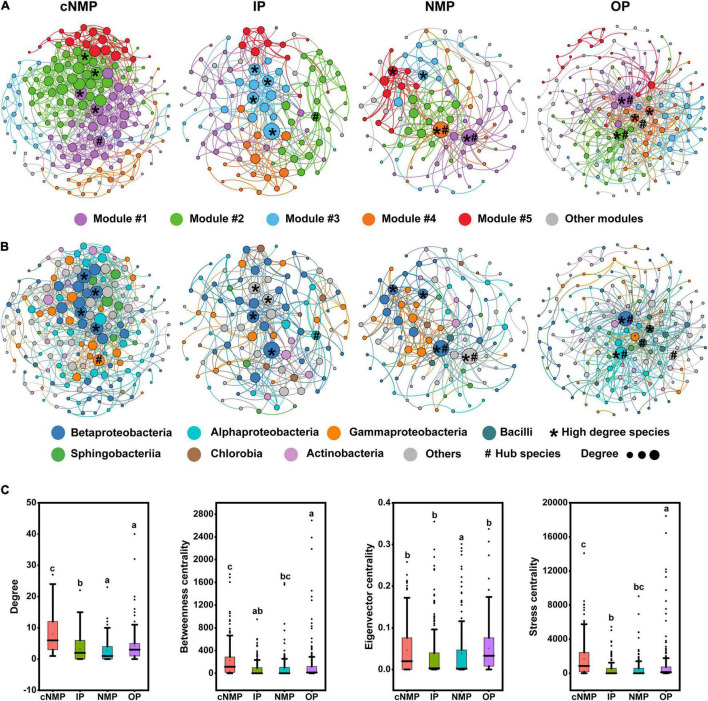
The network was established by calculating correlations among microbial community under the influence of four kinds of phosphorus sources. The nodes of the network are colored according to **(A)** ecological clusters (modules) and **(B)** classification of bacteria at the class level. Nodes represent OTUs. The node size is proportional to the average degree, and links between nodes indicate significant correlations. Modules are randomly colored at each phosphorus source. **(C)** Comparison of node-level topological features among four different communities. Higher-degree species means the top four nodes with the highest links. Hub species means key species through connectivity calculation. The top and bottom boundaries of each box represent the 75th and 25th quartile values, respectively; and the line within each box represents the median values. Different letters indicate significant differences at the *p* < 0.05 level. OTU, operational taxonomic unit.

Identifying the interactions occurring among different microorganisms and among the keystone species is essential for better understanding the microbial community diversity and functions. Four pMENs (*S*_*t*_ = 0.84) were constructed to analyze the co-occurrence patterns of bacterial communities in DPR under four phosphorus sources (Supplementary Table 6 and Figure 5). All the connectivity distributions followed the power-law model (power-law *R*^2^ from 0.557 to 0.858), indicating that relatively few nodes had multiple connections, and most nodes have fewer connections. The modularity values of all co-occurrence networks ranged from 0.455 to 0.516, which was significantly higher than the corresponding random network (0.287 to 0.423). These empirical networks exhibited network topology properties, such as scale-free, modularity, and small-world (Deng et al., 2012). The community interactions in cNMP-containing samples were more stable with the most complex network (avgK, 8.012; avgCC, 0.394) (Figure 5A and Supplementary Table 6), which was followed by the complexity of network observed in IP-containing sample (avgK, 5.545; avgCC, 0.304) (Figures 5A,B and Supplementary Table 6). The unique node-level topological features of the four communities were compared. It was observed that as compared with IP samples, the degree, betweenness centrality, and stress centrality values of cNMP samples’ OTUs were higher, while the degree values of NMP and OP samples’ OTUs were lower than those of IP samples. Also, the stress centrality of OP sample and the eigenvector centrality values of NMP sample was also lower than that of IP samples (Figure 5E). These results indicated that cNMP community network interactions were much more intense.

The bacteria represented by the four nodes with the highest average connectivity in each network were closely related to the denitrifying nitrogen and phosphorus removal bacteria, such as *Candidatus_*Accumulibacter, *Dechloromonas*, *Pseudomonas*, and *Nitrospira* (Sidat et al., 1999; Figure 5; and Supplementary Table 7). The four nodes in cNMP sample belonged to the abundant taxa, whose average abundance was greater than 0.1% (Liu et al., 2015) (Supplementary Table 7). The Z-P plot showed one module hub in cNMP samples, one module hub in IP samples, one module and three network hubs in OP samples, and one module hub and one network hub in NMP samples (Supplementary Figure 2). These results demonstrated that the overall module structures of the four networks were extremely dependent on the particular bacterial species. In addition, no keystone taxa were shared in four networks at the OTU and genus levels, as shown in Supplementary Table 8. The average abundance of all keystone taxa was less than 0.1%, except for the module hub in IP samples, indicating that they belonged to rare taxa. It is worth noting that the module hub in IP samples belonged to *Rhizobium*_*radiobacter* (average abundance 5.79%), which is related to denitrification (Dandie et al., 2007). This indicated that some low-abundant species might play important roles in shaping community structures.

### Prediction of the Functional Pathways and Potential Functions of the Denitrifying Phosphorus Removal Microbial Community

The potential functional characteristics of the microbial community of cNMP-, IP-, NMP-, and OP-containing samples were predicted using PICRUSt2, based on the 16S high-throughput sequencing and KEGG database. Although the species richness and diversity of cNMP-containing samples were higher than those of IP-containing samples, the functional richness and diversity of the former were significantly lower than those of the latter (Supplementary Figure 3). The predicted genes were divided into four major functional groups of the genes: cellular processes (7.46–8.97%), environmental information processing (2.88–3.19%), genetic information processing (7.47–7.71%), and metabolism (72.07–74.12%) (Supplementary Figure 4A). The most abundant gene in the metabolism category belonged to amino acid metabolism (11.81–12.05%), followed by carbohydrate metabolism (8.51–8.99%) and metabolism of cofactors and vitamins (8.09–8.64%). The relative abundance of carbohydrate metabolism and lipid metabolism genes in the cNMP-containing samples was higher than in the other three phosphorus groups (Supplementary Figure 4A). This indicated that cNMPs promoted the enhancement of carbohydrate and lipid metabolism in the microbial community. Based on KEGG database, energy metabolism subgroups were further analyzed (Supplementary Figures 4B,C). In the enhanced biological phosphorus removal (EBPR), poly-P kinase (PPK) and poly-P exopolyphosphatase (PPX) are two important enzymes (Guo et al., 2017), where PPK is responsible for the synthesis of poly-P from ATP in PAOs (Burow et al., 2007). On the other hand, PPX catalyzes the removal of the terminal phosphate from poly-P (Lee et al., 2006). The average relative abundance of PPX in the cNMP-containing sample was 1.31‰, which was higher than the relative abundance of 1.00‰ observed for IP-containing sample. In addition, the main nitrogen removal pathways in the system were also investigated (Supplementary Figure 4C). Denitrification-related enzymes were detected in the sludge samples, and it was observed that the relative abundance of nitrate reductase, nitrite reductase, nitric-oxide reductase, and nitrous-oxide reductase was higher in cNMP-, NMP-, and OP-containing sample than in IP-containing sample.

## Discussion

This study combined ecological null model, network analysis, and PICRUSt2 to reveal the community assembly mechanism, bacterial network structure, and functional metabolic pathway under different phosphorus sources, for the first time. The results showed that the characteristics of bacterial community (taxonomic diversity and topological characteristics of symbiotic networks), the community assembly mechanism (bacterial proportion affected by deterministic or stochastic processes), and the main functional metabolic pathways changed under different phosphorus sources. According to these results, it was proposed that the microbial communities under the impact of different phosphorus sources exhibit distinct ecological patterns and functions that are driven by different bacterial assembly processes.

### Dependence of Denitrifying Phosphorus Removal Bacterial Community Assembly on Phosphorus Sources

Deterministic factors like environmental filtering (e.g., carbon sources and nutrients) are responsible for driving the microbial community construction in a bioreactor. In the current study, all the four types of bacterial communities were regulated by different phosphorus sources (cNMPs, IPs, NMPs, and OPs), which support the vital role of homogeneous selection in the formation of bacterial communities (Figure 4 and Supplementary Table 4). In the sample community contain four phosphorus sources, the trend observed with respect to the effect of deterministic process was completely opposite to the trend observed with bacterial diversity (Figure 2 and Supplementary Figure 3). This further supported the important role of environmental selection in the construction of a bacterial community (Zhang et al., 2019). Among the pMENs of these bacterial microbiomes, most species of higher connectivity belonged to DPAOs; this indicated that these keystone species showed strong habitat filtering characteristics. This was consistent with other bacterial community strongly influenced by environmental conditions (e.g., availability of substrates and resources, redox potential) (Xu et al., 2019). Meanwhile, the keystone species of IP-containing sample (*Rhizobium_radiobacter*) was DPAOs (Dandie et al., 2007), and it was the only species that belonged to the abundant taxa (average relative abundance 5.787%) (Supplementary Table 8). Rare taxa are more vulnerable to ecological drift, because even a small decline in their number can result in species extinction (Fodelianakis et al., 2021). The abundance of key species in IP-containing sample was much higher than that in the other three groups, which may lead to the highest deterministic process.

In addition, unlike in IP-containing samples, the increase in bacterial community diversity in the cNMP-containing sample was consistent with the increase in the proportion of stochastic processes (e.g., dispersal limitation) and network complexity (Figure 4 and Supplementary Table 6). Li et al. (2021) also reported the same connection between bacterial diversity, network structures, and assembly in full-size biologically activated carbon filters under ozone implementation processes. The distribution of species in the microbial community of samples containing IPs was more concentrated than in the samples containing cNMPs. This resulted in the lower diversity and uneven distribution of community composition in IP-containing samples (Figure 2). Under this single-species structure, the network structure formed was simple, and the metabolic interaction between the taxa was weaker than observed in case of samples containing cNMPs (lower clustering coefficient and average connectivity are presented in Supplementary Table 6).

The microbial communities affected by the two phosphorus sources of IPs and cNMPs showed different species interaction levels and community distribution patterns, which also supports the null model result; thus, the uneven distribution of species is more likely to be dominated by deterministic process, and community with higher species diversity and strong species interaction always dominated by stochastic processes (Fuhrman, 2009; Lin et al., 2017).

Neutral dynamics and deterministic dynamics operate simultaneously in the process of bacterial community assembly, and their balance depends on the richness of the initial community (Ayarza and Erijman, 2011; Zhang et al., 2020). Under the condition of high microbial diversity, the process of stochastic assembly of the community dominated. However, under the condition of low microbial diversity, the process of deterministic assembly of the community dominated (Xun et al., 2019). The variation trend of determinism was exactly opposite to the DPR bacterial diversity, further supporting the important role of environmental selection in the assembly of bacterial community (Zhang et al., 2019; Xu et al., 2020). The microbial diversity under the action of inorganic phosphorus was the lowest, so microbial groups tend to “fight with each other,” and the selection of community maintenance conditions or homogenization is a deterministic assembly process (Rendueles and Velicer, 2017). Thus, the final space can only be occupied by a few dominant species. The microbial diversity of bacterial species under the impact of cNMPs was higher than under the impact of IPs. Thus, the community under cNMPs can hold and express more specific functions and adapt to the environment, compared with the community under the impact of IPs, and can maintain a stochastic assembly process with random growth, reproduction, and death (Ofiteru et al., 2010; Dini-Andreote et al., 2015).

### Response of Microbial Species and Functional Diversity Driven by Phosphorus Source

The microbial community is the core contributor to the function of the ecosystem, especially the pollutant-degrading bacterial community used in this study (Miki et al., 2010). Many coexisting but taxonomically different microorganisms can encode the same metabolic function, which is in sharp contrast to the expectation that species should occupy different metabolic niches (Jung et al., 2016; Louca et al., 2018). Sensitive taxa are easily extinct or dormant due to interference from the external environment. Functionally redundant taxa can buffer the adverse effects of such interference on the microbial community and enable the tolerant taxa to continue to perform corresponding ecological functions (Galand et al., 2018). The results from current study showed that under the impact of cNMPs, the highest species diversity and lowest functional diversity was observed in the system (Figure 2A and Supplementary Figure 2). This implies that the communities showed a high degree of “functional redundancy”; i.e., each metabolic function can be performed by a variety of coexisting and taxonomically different organisms. According to Supplementary Figure 4C, the functional redundancy in samples containing cNMPs was mainly related to the nitrogen and phosphorus metabolism, which indicates that cNMPs are more suitable for nitrogen and phosphorus removal by denitrification. In addition, the high species diversity under the impact of cNMPs might be because the dephosphorylated cNMPs provided inorganic phosphorus and additional carbon sources, which caused the microbial community to benefit from the ideal nutrient availability and environmental conditions, supporting bacterial growth and diversity (Xie et al., 2021).

Summarizing, the results of functional prediction showed that the changes in the metabolism subcategories, including energy metabolism, carbohydrate metabolism, and lipid metabolism, between all the samples were not statistically significant (*p* > 0.05). However, the results indicated the metabolic divergence of phosphorus and nitrogen metabolism. As compared with the samples containing IPs, the predicted exopolyphosphatase, nitrate reductase, and nitrite reductase enzymes in biofilm samples containing cNMPs were increased. This implies the potential overcapacity of functional enzymes (Cui et al., 2019), which allows for lower residual phosphorus concentration in cNMP-containing samples. In addition, the bacteria represented by the four nodes with the highest average connectivity in cNMP-containing samples were DPR bacteria, and their relative abundance was higher than 0.1%, indicating abundant taxa. It was indicated that the presence of cNMPs stimulated the enrichment of DPR bacteria and the expression of functional genes related to nitrogen and phosphorus removal. The corresponding enzyme activity was enhanced, and the content of residual phosphorus and nitrogen in the system was finally reduced (Huang et al., 2019). Thus, cNMPs can be considered as the best favorite phosphorus source with respect to the DPR microbial community structure, although IPs are the only phosphorus source that can be directly utilized by microorganisms.

## Conclusion

The relationship between community assembly mechanism, microbial interactions, and functions of DPR sludge system was investigated under impact of four kinds of phosphorus sources (IPs, cNMPs, NMPs, and OPs), under the same environment and operation conditions. The main conclusions of this work are summarized as follows:

(1) Phosphorus could mediate either deterministic or stochastic process in DPR system. Determinism dominated in IP-influenced community assembly, while stochasticity dominated in cNMP-influenced community assembly.
(2) The cNMPs can be considered as the best favorite phosphorus source for microorganism, and species interactions were more intensive in cNMP-influenced microbiota.
(3) The cNMPs increased the activity of enzymes related to denitrification and phosphorus metabolism and increased the α-diversity of microorganism but decreased the diversity of metabolic function.

## Data Availability Statement

The datasets presented in this study can be found in online repositories. The names of the repository/repositories and accession number(s) can be found in the article/Supplementary Material.

## Author Contributions

LZ: conceptualization, methodology, writing- reviewing and editing, and funding acquisition. XW: software, validation, formal analysis, and writing-original draft. AD: writing-reviewing and editing. DY: validation and formal analysis. QT: software and validation. YX: visualization and investigation. EX: conceptualization, resources, writing- reviewing and editing, and funding acquisition. All authors contributed to the article and approved the submitted version.

## Conflict of Interest

The authors declare that the research was conducted in the absence of any commercial or financial relationships that could be construed as a potential conflict of interest.

## Publisher’s Note

All claims expressed in this article are solely those of the authors and do not necessarily represent those of their affiliated organizations, or those of the publisher, the editors and the reviewers. Any product that may be evaluated in this article, or claim that may be made by its manufacturer, is not guaranteed or endorsed by the publisher.

## Footnotes

1
huttenhower.sph.harvard.edu/galaxy

2
ieg4.rccc.ou.edu/mena

3
ieg3.rccc.ou.edu:8080/
