# Supplementary material for: Ecological Insights Into Community Interactions, Assembly Processes and Function in the Denitrifying Phosphorus Removal Activated Sludge Driven by Phosphorus Sources
Source: Front Microbiol. 2021 Nov 10;12:779369. doi: 10.3389/fmicb.2021.779369 (PMC8660105; doi:10.3389/fmicb.2021.779369)
Supplement: Supplementary file 1 [file Data_Sheet_1.docx]

**Supplementary Information**

For

**Ecological insights into community interactions, assembly processes and function** **in the denitrifying phosphorus removal activated sludge driven by phosphorus sources**

**Lei Zheng^1^, Xue Wang^1^, Aizhong Ding^1^, Dongdan Yuan^1^, Qiuyang Tan^1^, Yuzi Xing^1^, En Xie^2*^**

1 College of Water Science, Beijing Normal University, Beijing 100875, China

2 College of Water Resources and Civil Engineering, China Agricultural University, Beijing 100083, China

*** Correspondence:**

EnXie, Ph.D.

Tel: +86(0)10-62736533

Email: xe@cau.edu.cn

Number of pages: 16

Number of tables: 8

Number of figures: 4

**Supplementary Methods**

**Method S1**

**The SBR reactor operation pattern**

The SBR reactor had a volume of 10 L and ran with a cycle time of 12 h, and 2 cycles per day and the volume exchange ratio was 60%. The SBR operation pattern was consisted of feeding (30 min), anaerobic (150 min), anoxic (180 min), aerobic (270 min), settlement (60 min) and decanting (30 min). The hydraulic retention time (HRT) of the SBR reactor was 18 h with a sludge retention time (SRT) of 7 days.

**Method S2**

**M9 medium**

The M9 medium were prepared as previously reported method(Akhtar and Rehman, 2017) with modification: C_6_H_12_O_6_·H_2_O (5.79 g/L), KCl (3.51 g/L), MgSO_4_ (0.98 g/L), FeSO_4_·7H_2_O (0.01 g/L), MnSO_4_·H_2_O (0.005 g/L), NaNO_3_ (1.275 g/L). Then adjusted to pH approximately 7.0 and autoclaved.

**Table caption**

**Table S1Contents of influent wastewater.**

**Table S2 59 phosphorus sources and their corresponding groups**

**Table S3P values of permutational multivariate analysis of variance (PERMANOVA) tests for bacterial community compositions (at OTU level) by PCoA based on the Bray-Curtis dissimilarity matrix.**

**Table S4P values of permutational multivariate analysis of variance (PERMANOVA) tests for bacterial community assembly in different phosphorus.**

**Table S5 The taxonomy information of 12 deterministic-dominated bins.**

**Table S6Topological properties of the empirical pMENs of microbial communities in each group and their associated random pMENs.**

**Table S7Taxonomic information and average abundance of the top four nodes with the highest connectivity taxa in each group observed in ecological networks in DPR communities sourced from different phosphorus sources.**

**Table S8Taxonomic information and average abundance of keystone taxa observed in ecological networks in DPR communities sourced from different phosphorus sources.**

**Figure caption**

**Fig. S1 Rarefaction curves of similarity-based operational taxonomic unit (OTU) at 97% sequence similarity level.**

**Fig. S2 Zi-Pi plot showing the distribution of OTUs based on their topological roles.**

**Fig. S3Function diversity characterized by the Equitability, Richness, Shannon and Simpson index.**

**Fig. S4PICRUSt2 prediction based on the KEGG database.**

**Table S1.** Effluent quality and removal efficiency in SBR.

| **Water quality indices** | **Concentration (mg/L)** | **Removal efficiency** |
| --- | --- | --- |
| **TOC** | 50.46 ± 1.32 | 81.7% |
| **NH_3_-N** | 6.48 ± 0.48 | 86.8% |
| **Totalnitrogen** | 8.82 ± 0.41 | 82.9% |
| **Total phosphorus** | 0.53 ± 0.07 | 89.3% |

**Table S2**59 phosphorus sources and their corresponding groups.

| **Phosphorus sources** | **group** | **Phosphorus sources** | **group** |
| --- | --- | --- | --- |
| Adenosine-2',3'-cyclic monophosphate | cNMPs | Triethyl phosphate | OPs |
| Adenosine-3',5'-cyclic monophosphate | cNMPs | D,L-α-glycerol phosphate | OPs |
| Guanosine-2',3'-cyclic monophosphate | cNMPs | β-glycerol phosphate | OPs |
| Guanosine-3',5'-cyclic monophosphate | cNMPs | Carbamyl phosphate | OPs |
| Cytidine-2',3'-cyclic monophosphate | cNMPs | D-2-phospho-glyceric acid | OPs |
| Cytidine-3',5'-cyclic monophosphate | cNMPs | D-3-phospho-glyceric acid | OPs |
| Uridine-2',3'-cyclic monophosphate | cNMPs | Phosphoenol pyruvate | OPs |
| Uridine-3',5'-cyclic monophosphate | cNMPs | Phospho-glycolic acid | OPs |
| Thymidine-3',5'-cyclic monophosphate | cNMPs | D-glucose-1-phosphate | OPs |
| Phosphate | IPs | D-glucose-6-phosphate | OPs |
| Pyrophosphate | IPs | 2-deoxy-D-glucose-6-phosphate | OPs |
| Trimeta-phosphate | IPs | D-glucosamine-6-phosphate | OPs |
| Tripoly-phosphate | IPs | 6-phospho-glucomic acid | OPs |
| Hypophosphite | IPs | D-mannose-1-phosphate | OPs |
| Thiophosphate | IPs | D-mannose-6-phosphate | OPs |
| Dithiophosphate | IPs | Cysteamine-S-phosphate | OPs |
| Adenosine-2'-monophosphate | NMPs | Phospho-L-arginine | OPs |
| Adenosine-3'-monophosphate | NMPs | O-phospho-D-serine | OPs |
| Adenosine-5'monophosphate | NMPs | O-phospho-L-serine | OPs |
| Guanosine-2'-monophosphate | NMPs | O-phospho-L-threonine | OPs |
| Guanosine-3'-monophosphate | NMPs | O-phospho-D-tyrosine | OPs |
| Guanosine-5'-monophosphate | NMPs | O-phospho-L-tyrosine | OPs |
| Cytidine-2'-monophosphate | NMPs | Phosphocreatine | OPs |
| Cytidine-3'-monophosphate | NMPs | Phosphoryl choline | OPs |
| Cytidine-5'-monophosphate | NMPs | O-phosphoryl-ethanolamine | OPs |
| Uridine-2'-monophosphate | NMPs | Phosphono acetic acid | OPs |
| Uridine-3'-monophosphate | NMPs | 2-aminoethyl phosphonic acid | OPs |
| Uridine-5'-monophosphate | NMPs | Methylene diphosphonic acid | OPs |
| Thymidine-3'-monophosphate | NMPs | Inositol hexaphosphate | OPs |
| Thymidine-5'-monophosphate | NMPs |  |  |

**Table S3**P values of permutational multivariate analysis of variance (PERMANOVA) tests for bacterial community compositions (at OTU level) by PCoA based on the Bray-Curtis dissimilarity matrix.

|  | IPs | OPs | NMPs | cNMPs |
| --- | --- | --- | --- | --- |
| IPs |  |  |  |  |
| OPs | 0.142 |  |  |  |
| NMPs | **0.047** | 0.101 |  |  |
| cNMPs | **0.001** | **0.005** | **0.016** |  |

Bold font means *p*<0.05.

**Table S4**P values of permutational multivariate analysis of variance (PERMANOVA) tests for bacterial community assembly in different phosphorus.

**(A) Heterogeneous selection**

|  | cNMPs | IPs | NMPs | OPs |
| --- | --- | --- | --- | --- |
| cNMPs |  |  |  |  |
| IPs | 0.173 |  |  |  |
| NMPs | 0.282 | 0.300 |  |  |
| OPs | 0.236 | 0.615 | 0.365 |  |

**(B) Homogeneous selection**

|  | cNMPs | IPs | NMPs | OPs |
| --- | --- | --- | --- | --- |
| cNMPs |  |  |  |  |
| IPs | **0.028** |  |  |  |
| NMPs | **0.041** | 0.316 |  |  |
| OPs | **0.042** | 0.218 | 0.369 |  |

**(C) Dispersal limitation**

|  | cNMPs | IPs | NMPs | OPs |
| --- | --- | --- | --- | --- |
| cNMPs |  |  |  |  |
| IPs | **0.005** |  |  |  |
| NMPs | **0.021** | 0.162 |  |  |
| OPs | **0.038** | 0.058 | 0.271 |  |

**(D) Homogenizing dispersal**

|  | cNMPs | IPs | NMPs | OPs |
| --- | --- | --- | --- | --- |
| cNMPs |  |  |  |  |
| IPs | 0.474 |  |  |  |
| NMPs | 0.601 | 0.562 |  |  |
| OPs | 0.421 | 0.407 | 0.343 |  |

**(E) Driftand others**

|  | cNMPs | IPs | NMPs | OPs |
| --- | --- | --- | --- | --- |
| cNMPs |  |  |  |  |
| IPs | 0.420 |  |  |  |
| NMPs | 0.390 | 0.517 |  |  |
| OPs | 0.372 | 0.524 | 0.512 |  |

**(F) Stochasticity**

|  | cNMPs | IPs | NMPs | OPs |
| --- | --- | --- | --- | --- |
| cNMPs |  |  |  |  |
| IPs | **0.036** |  |  |  |
| NMPs | 0.053 | 0.320 |  |  |
| OPs | 0.058 | 0.211 | 0.357 |  |

Bold font means *p*< 0.05.

**Table S5The taxonomy information of 12 deterministic-dominated bins**

| **Bin** | **Domain** | **Phylum** | **Class** | **Order** | **Family** | **Genus** |
| --- | --- | --- | --- | --- | --- | --- |
| Bin3 | *Bacteria* | *Planctomycetes* | *Phycisphaerae* | *Phycisphaerales* | *Phycisphaeraceae* | *SM1A02* |
| Bin7 | *Bacteria* | *Firmicutes* | *Clostridia* | *Clostridiales* | *Lachnospiraceae* | *Eubacterium_rectale_group* |
| Bin14 | *Bacteria* | *Actinobacteria* | *Thermoleophilia* | *Gaiellales* | *unidentified* | *unidentified* |
| Bin19 | *Bacteria* | *Gemmatimonadetes* | *Longimicrobia* | *Longimicrobiales* | *Longimicrobiaceae* | *unidentified* |
| Bin22 | *Bacteria* | *Acidobacteria* | *Blastocatellia* | *Blastocatellales* | *Blastocatellaceae_Subgroup_4* | *unidentified* |
| Bin27 | *Bacteria* | *Proteobacteria* | *Alphaproteobacteria* | *Rhizobiales* | *Unassigned* | *Unassigned* |
| Bin30 | *Bacteria* | *Proteobacteria* | *Betaproteobacteria* | *Burkholderiales* | *Comamonadaceae* | *Variovorax* |
| Bin31 | *Bacteria* | *Proteobacteria* | *Betaproteobacteria* | *Rhodocyclales* | *Rhodocyclaceae* | *Candidatus_Accumulibacter* |
| Bin32 | *Bacteria* | *Proteobacteria* | *Gammaproteobacteria* | *Pseudomonadales* | *Pseudomonadaceae* | *Pseudomonas* |
| Bin38 | *Bacteria* | *Bacteroidetes* | *Bacteroidia* | *Bacteroidales* | *Rikenellaceae* | *Alistipes* |
| Bin40 | *Bacteria* | *Bacteroidetes* | *Bacteroidia* | *Bacteroidales* | *Bacteroidaceae* | *Bacteroides* |
| Bin41 | *Bacteria* | *Bacteroidetes* | *Sphingobacteriia* | *Sphingobacteriales* | *Chitinophagaceae* | *Ferruginibacter* |

**Table S6**Topological properties of the empirical pMENs of microbial communities in each group and their associated random pMENs.

|  | Real networks | | | | | | |  | Random networks | | |
| --- | --- | --- | --- | --- | --- | --- | --- | --- | --- | --- | --- |
|  | Similarity  threshold (St) | R^2^ | Nodes | Average connectivity  (avgK) | Average path length  GD | Average clustering coefficient（avgCC） | Modularity  (No. of modules) |  | Average path length (Zhang et al.) | Average clustering coefficient  (avgCC) | Modularity |
| cNMPs | 0.84 | 0.704 | 171 | 8.012 | 3.831 | 0.394 | 0.490(6) |  | 2.772 ± 0.026 | 0.090 ± 0.011 | 0.287 ± 0.007 |
| IPs | 0.84 | 0.557 | 101 | 5.545 | 3.589 | 0.304 | 0.491(6) |  | 2.882 ± 0.036 | 0.081 ± 0.015 | 0.353 ± 0.011 |
| NMPs | 0.84 | 0.780 | 105 | 4.305 | 3.842 | 0.174 | 0.516(8) |  | 3.131± 0.062 | 0.092 ± 0.017 | 0.416 ± 0.009 |
| OPs | 0.84 | 0.858 | 163 | 4.405 | 3.518 | 0.064 | 0.455(11) |  | 3.183 ±0.048 | 0.087 ± 0.012 | 0.423 ± 0.008 |

**Table S7** Taxonomic information and average abundance of the top four nodes with the highest connectivitytaxa in each group observed in ecological networks in DPR communities sourced from different phosphorus sources.

| Network | OTU | degree | Lowest taxonomic rank | Affiliate Phylum | Average abundance(%) |
| --- | --- | --- | --- | --- | --- |
| cNMPs | OTU_26 | 27 | g__*Candidatus*_Accumulibacter | *Proteobacteria* | **0.294** |
|  | OTU_509 | 24 | g__*Candidatus*_Accumulibacter | *Proteobacteria* | **0.392** |
|  | OTU_2 | 24 | g__*Candidatus*_Accumulibacter | *Proteobacteria* | **1.642** |
|  | OTU_118 | 23 | g__*Dechloromonas* | *Proteobacteria* | **0.284** |
| IPs | OTU_509 | 22 | g__*Candidatus*_Accumulibacter | *Proteobacteria* | 0.056 |
|  | OTU_10 | 15 | g__*Nitrospira* | *Nitrospirae* | 0.066 |
|  | OTU_26 | 15 | g__*Candidatus*_Accumulibacter | *Proteobacteria* | 0.091 |
|  | OTU_38 | 14 | o__*MVP-88* | *Elusimicrobia* | 0.008 |
| NMPs | OTU_3579 | 23 | f__*Comamonadaceae* | *Proteobacteria* | 0.006 |
|  | OTU_149 | 23 | f__*Blastocatellaceae_Subgroup_4* | *Acidobacteria* | 0.003 |
|  | OTU_118 | 13 | g__*Dechloromonas* | *Proteobacteria* | 0.062 |
|  | OTU_2 | 13 | g__*Candidatus*_Accumulibacter | *Proteobacteria* | **0.426** |
| OPs | OTU_2113 | 40 | g__*Chitinimonas* | *Proteobacteria* | 0.002 |
|  | OTU_897 | 32 | g__*Kaistia* | *Proteobacteria* | 0.007 |
|  | OTU_3590 | 20 | g__*Pseudomonas* | *Proteobacteria* | 0.002 |
|  | OTU_1013 | 19 | g__*Lactobacillus* | *Firmicutes* | 0.004 |

*Bold font means average abundance was more than 0.1%.

**Table S8**Taxonomic information and average abundance of keystone taxa observed in ecological networks in DPR communities sourced from different phosphorus sources.

| Network | OTU | Topological position | Lowest taxonomic rank | Affiliate Phylum | Average abundance(%) |
| --- | --- | --- | --- | --- | --- |
| cNMPs | OTU_70 | Module hub | g__*Candidatus*_Competibacter | *Proteobacteria* | 0.054 |
| IPs | OTU_9 | Module hub | s__*Rhizobium*_radiobacter | *Proteobacteria* | **5.787** |
| NMPs | OTU_149 | Module hub | f__*Blastocatellaceae_Subgroup_4* | *Acidobacteria* | 0.003 |
|  | OTU_3579 | Network hub | f__*Comamonadaceae* | *Proteobacteria* | 0.006 |
| OPs | OTU_245 | Module hub | c__*1-20* | *Chloroflexi* | 0.003 |
|  | OTU_897 | Network hub | g__*Kaistia* | *Proteobacteria* | 0.007 |
|  | OTU_988 | Network hub | g__*Oenococcus* | *Firmicutes* | 0.003 |
|  | OTU_2113 | Network hub | g__*Chitinimonas* | *Proteobacteria* | 0.002 |

*Bold font means average abundance was more than 0.1%.


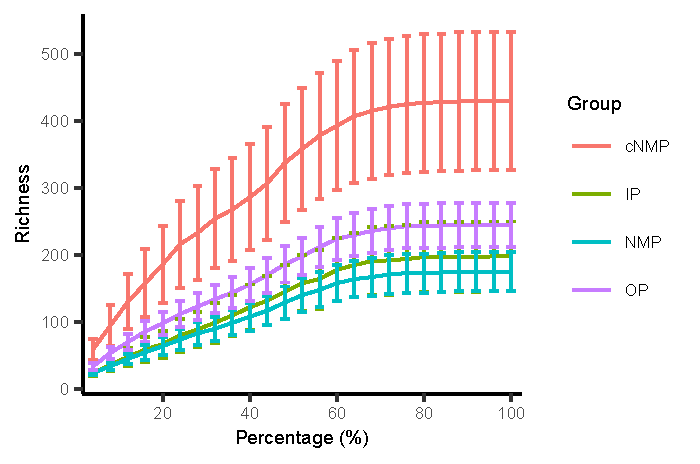


**Fig. S1** Rarefaction curves of similarity-based operational taxonomic unit (OTU) at 97% sequence similarity level.


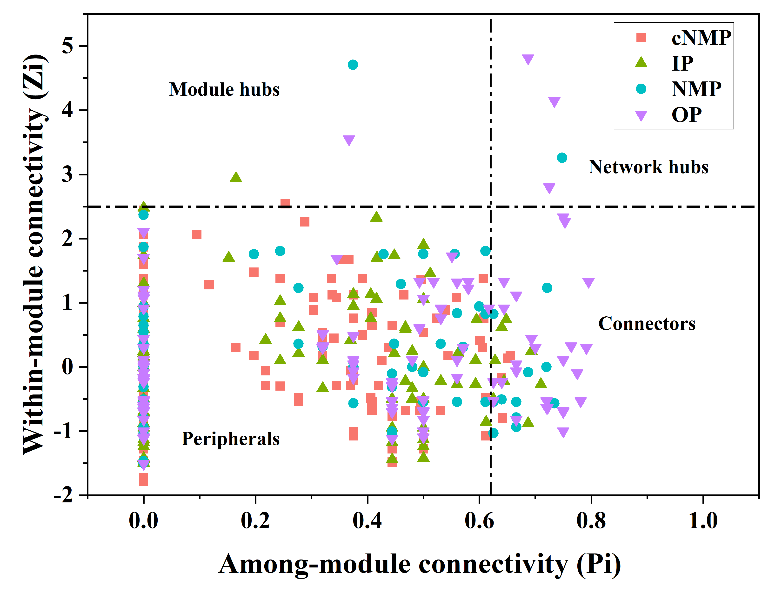


**Fig. S2** Zi-Pi plot showing the distribution of OTUs based on their topological roles. Each symbol represents an OTU. The topological role of each OTU was determined according to the scatter plot of within-module connectivity (*Zi*) and among-module connectivity (*Pi*).

**
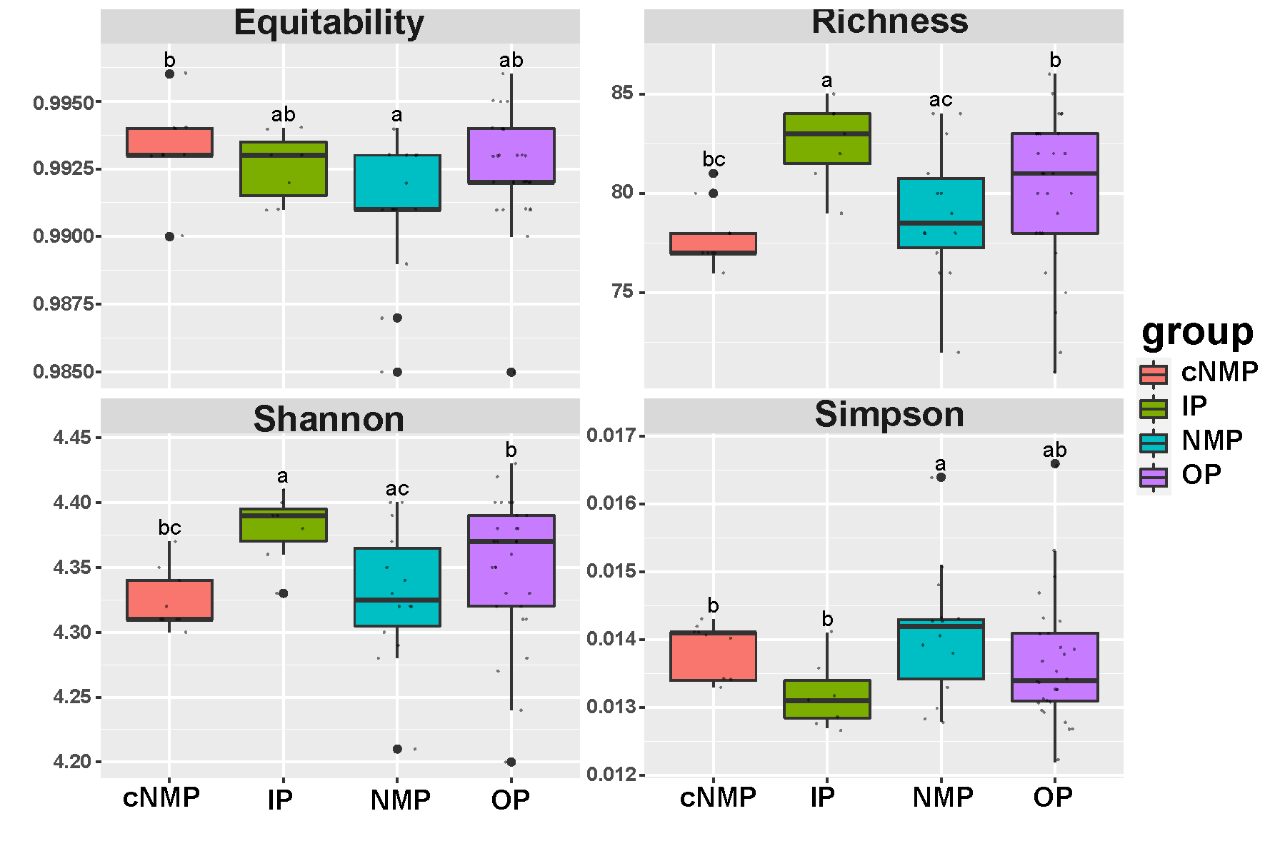
**

**Fig. S3** Function diversity characterized by the Equitability, Richness, Shannon and Simpson index.


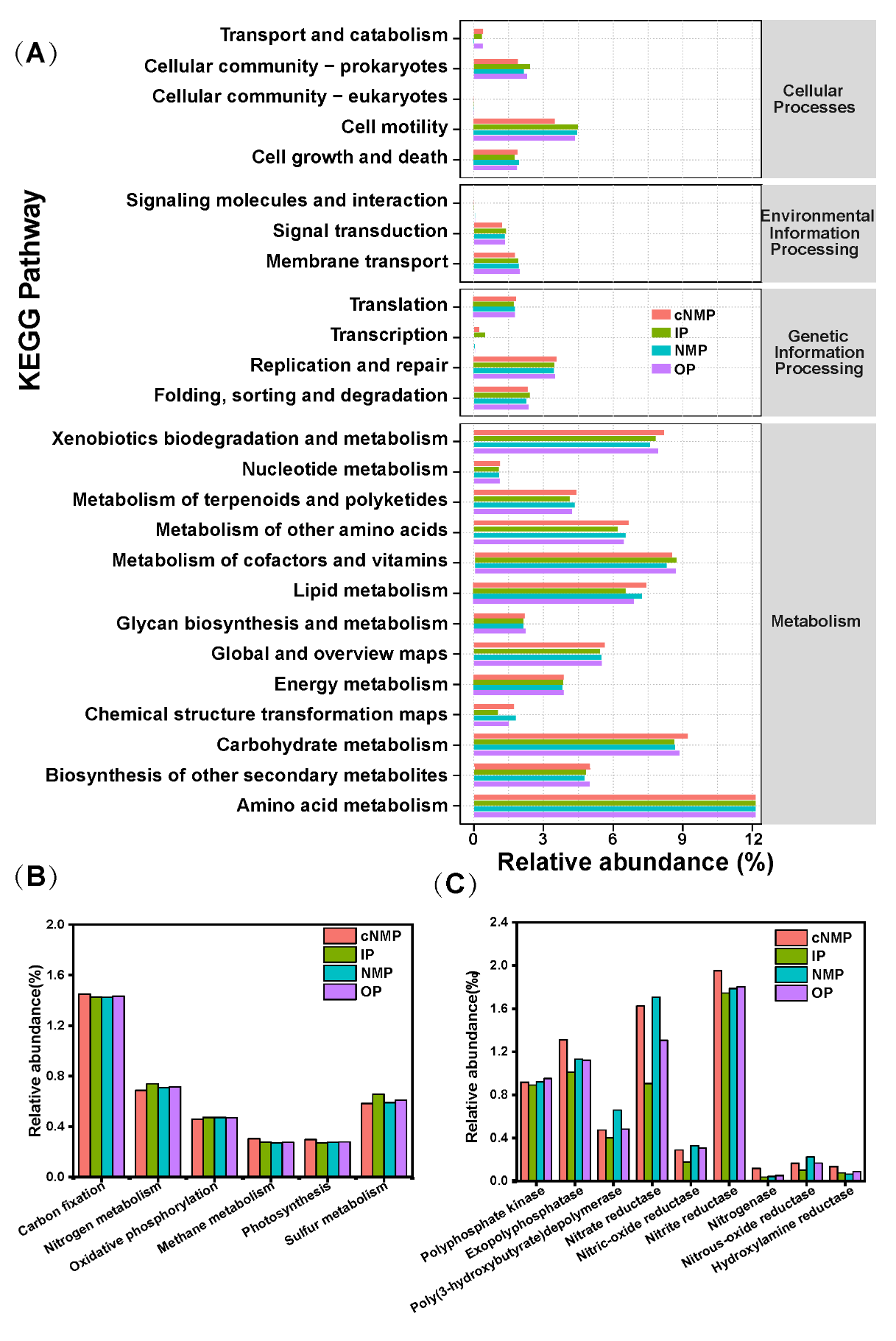


**Fig. S4**PICRUSt2 prediction based on the KEGG database: (A) function prediction; (B) analysis on the subsystem of energy production and conversion; (C) enzyme analysis on phosphorus and nitrogen metabolism.

**References**

Akhtar, A., and Rehman, A. (2017). Tellurite reduction potential of bacteria isolated from industrial wastewater. *Punjab Univ. J. Zool* 32**,** 129-135.

Zhang, M., Zhu, C., Pan, T., Fan, Y., Liu, Y., He, C., et al. (2020). Elucidating sludge characteristic, substrate transformation and microbial evolution in a two-sludge denitrifying phosphorus removal system under the impact of HRT. *Journal of Environmental Management* 262. doi: 10.1016/j.jenvman.2020.110391.
